# The Royal Hospital for Incurables

**Published:** 1916-04-15

**Authors:** 

**Affiliations:** President of the Ladies' Association.


### The Royal Hospital for Incurables.

Letter from H.R.H. Princess Christian, President of the Ladies' Association.

*To the Editor of* The Hospital.

Sir,—There is no sadder word in the English language than "incurable," no word that in our own case we should more dread to hear, yet every year adds a certain number to this terrible category, and the indirect results of the war will undoubtedly add many more. Amongst the innumerable cases which apply for help to the Royal Hospital for Incurables, Putney Heath, some are almost entirely unbefriended, and have therefore little or no hope of securing election. It is with such cases that the Ladies' Association of the Royal Hospital for Incurables specially deals. In the years before the war an annual entertainment was given to raise money before the May election. Such an entertainment is at present out of the question, but we appeal with confidence to the public to help us to find the sum of £500 with which to alleviate the sufferings of a few of those whose life is at best but a prolonged martyrdom. All subscriptions may be sent to the Hon. Secretary, Miss Allcroft, 93 Elizabeth Street, Eaton Square, S.W.

Helena,
President of the Ladies' Association.

April 11, 1916.

